# Judgments of relevance in preschoolers: a study of training and transfer of self-cueing strategies

**DOI:** 10.3389/fpsyg.2024.1341572

**Published:** 2024-01-30

**Authors:** Marion Leclercq, Wilfried T. Mombo, Jérôme Clerc

**Affiliations:** ^1^Univ Lille, ULR 4072 – PSITEC: Psychologie: Interactions, Temps, Emotions, Cognition, Composante INSPE Academie de Lille HdF, Lille, France; ^2^Univ Tours, EA 2114 – PAVeA: Psychologie des Âges de la Vie et Adaptation, Tours, France; ^3^Univ. Grenoble Alpes, Univ. Savoie Mont Blanc, CNRS, Grenoble, France

**Keywords:** goal identification, transfer, self-regulated learning, early childhood, self-cueing strategies

## Abstract

**Introduction:**

When facing a task, children must analyze it precisely to fully identify what its goal is. This is particularly difficult for young children, who mainly rely on environmental cues to get there. Research suggests that training children to look for the most relevant perceptual cues is promising. Furthermore, as transferring skills to a new task is difficult, the question of whether young children are able to transfer such training remains open. The aim of this study was to test the extent to which two strategies of goal self-cueing—labeling and pointing—can help 4-year-old children to identify the relevant cues to clearly identify the goal of the task. The effects of explicit strategy training were tested in a near transfer task.

**Method:**

Ninety-nine typically developing 4 year olds took part in the study. They were divided into three groups: two were trained collectively in one of the two strategies and the third group as a control group with no strategy training. All children performed a cued card-sorting task four times: Pre-test, Collective training, Post-test, and Transfer with new cards.

**Results:**

Results confirmed the beneficial effect of strategy training on goal identification, particularly after training (Post-test). In the transfer phase, all three groups performed equally well.

**Discussion:**

This study contributes to our understanding of how young children seek information when they look for the most relevant cues for identifying the goal of a task, and the benefits they may derive in a transfer task. It seems that the use of visual cues and self-cueing strategies helps preschoolers to clearly identify the goal of a task. Results are discussed in the light of the self-regulated learning framework. Some possible classroom applications are suggested.

## Highlights

- Positive effect of strategy training on the self-identification of the goal of the tasks is shown in 4-year-old children.
- Increased strategic and card-sorting performance occurs after training.
- Benefits of strategy training are maintained in transfer phase with new cards.
- Clarifying the goal of a task helps children to judge the relevance of information and to make consistent decisions.

## 1 Introduction

Learning to assess the relevance of information received in order to make informed decisions is a complex activity. Teachers can play a decisive role in helping students develop the necessary skills. An important first step may be to help students understand task instructions in order to identify the underlying goal of a school task. However, in the school context, the knowledge, skills and procedures needed to perform certain tasks are not systematically or sufficiently taught. Yet, instruction can improve students’ level of relevance, i.e., the capacity to identify any task goal relevant information. The relevance of information depends on decision-making and on the changing context. It evolves over time, and is often judged intuitively, without explicit reflection. This area remains studied scarcely in the lifelong learning context. The theoretical framework of self-regulated learning (SRL) allows us to focus specifically on the question of goal identification, and the use of cognitive strategies to achieve a learning goal.

SRL is made up of the processes by which learners mobilize knowledge and strategies according to the context, in order to organize and control their own learning in order to achieve their goals (Zimmerman, 2008; Schunk and Zimmerman, 2011; Winne, 2018). These processes build on the learner’s ability to reflect, adapt and adjust in the face of change, thus linking personal and contextual characteristics in a learning situation (Winne and Perry, 2000; Boekaerts and Corno, 2005; Cleary et al., 2012; Pino-Pasternak et al., 2014; Leclercq et al., 2023). The key skills for self-regulation consist in being able to (1) identify goals that give meaning to the work and set them as goals to be achieved, (2) plan one’s work and revise this plan if necessary, by adapting and modifying one’s behavior in the face of internal or environmental changes, and (3) protect oneself from distractions by adopting a wide variety of strategies (goal revision, organization, elaboration) which are necessary for carrying out academic tasks. This implies a final key skill which is (4) to be aware of the essential or recurring features of one’s behaviors, and to generate internal feedback about the learning process in order to be able to adjust and control learning (Butler and Winne, 1995; Pintrich, 2000, 2004; Winne, 2011, 2018; Gorges and Göke, 2015).

SRL thus enables us to better understand how learners form and maintain learning intentions, and how they can judge the relevance of information for the learning goal. Current work aims to better understand the factors that influence SRL, such as task and context characteristics (Cleary et al., 2012; Pino-Pasternak et al., 2014; Gorges and Göke, 2015; Leclercq et al., 2023). Developmental aspects of SRL have been studied in recent years, although they have long been ignored and largely underestimated in the youngest children (Zimmerman and Kitsantas, 1997; Montroy et al., 2016; Perry, 2019). However, the preschool period appears to be a sensitive time slot that supports many changes in language, thinking and reasoning (Garon et al., 2008; Jeong and Frye, 2020). The growth in information processing capacity during this period enables young children to better understand and perform many tasks due to an increasing capacity to self-regulate (Bronson, 2000; Perels et al., 2009; Perry, 2019). Current research seeks to clarify what the determinants and precursors of this complex process are, as early as the preschool period (Jacob et al., 2019; Zachariou and Whitebread, 2019; Dörr and Perels, 2020; Jeong and Frye, 2020).

There is a consensus on three existing phases in SRL (Winne, 2011, 2018; Zimmerman, 2013). It includes (1) a preparatory planning phase before engaging in the activity. After studying the perceived resources and task constraints, the learner can identify a goal and plan how to achieve it using appropriate strategies; (2) a commitment and follow-up phase during the activity, when the plan devised in phase 1 is implemented. Cognitive strategies are used to meet the demands of the task and help achieve the goal; (3) an evaluation phase once the activity has been completed, to update knowledge of the task and dedicated performance. This evaluation may lead to adjustments and modifications to the action plan used in phase 2, depending on constraints that may arise during the course of the action.

The first phase, encompassing goal identification and planning, is of great importance. Indeed, it is necessary to know where to go and what to do, to meet the demands of a task: one cannot achieve a goal without knowing how to get there. A goal is defined as “*what an individual is consciously trying to achieve*” (Schunk, 1990, p. 71). Goals are therefore crucial to the decision-making process. They have several important functions, such as eliminating unrealistic options or indicating what information to gather and what plans to develop to achieve them (Galotti, 2005). They are both the result to be achieved, and what guides action, giving it direction and energy. Goal identification is based on some cues available in the environment, which indicate the task, the procedure to be used, and the action to be taken (Chevalier et al., 2017; Blaye, 2021). The learner must deduce from the available information what the goal of the task is. Once the goal of the task has been identified thanks to the relevant information available in the environment and in the task itself, the learner enters the second phase of SRL. In this phase, cognitive strategies are required.

A cognitive strategy is a procedure or a set of procedures, produced intentionally to achieve a goal (Lemaire and Reder, 1999) and improve performance (Bjorklund et al., 1997). This includes learning strategies and self-regulatory strategies, which may be cognitive, metacognitive or volitional, and which are all closely linked to self-regulation processes. Taken together, the numerous strategies of an individual constitute its strategic repertoire. This repertoire can be impacted by training. Strategies are tools for learning which seem worthwhile to equip learners with. Strategies should therefore be taught (Winne, 1996). Furthermore, recent research suggests that learners should be asked to clarify their own expectations, make their goals explicit and record them in writing, and determine a work schedule for self-improvement (see Blaye, 2021, for a recent review).

The knowledge and skills required in a task are essential for identifying a goal, but so are the relevant cues available in the environment, which will guide goal identification. Moreover, goal identification and strategic engagement are difficult for young children (Blaye and Chevalier, 2011; Perry, 2019; Jeong and Frye, 2020). Their cognitive ability to consider cues in the environment is still developing, which means that they are not yet fully aware of all aspects of a situation. Making the goal explicit, in a very concrete way, by intervening directly on the environment to materially represent the goal, seems favorable. Furthermore, while the cues provided by a task appear to help young children improve their performance, the nature of these cues and their links with the task determine their relevancy and consequently, their effectiveness. The use of explicit self-cueing strategies seems fruitful with the youngest children, especially verbal labeling and pointing (Lucenet and Blaye, 2014, 2019).

The labeling strategy involves verbalizing the goal cue aloud. It thus provides an explicit and meaningful representation of the task goal. In the youngest children, strategic labeling is useful for identifying the goal of many tasks (Yerys and Munakata, 2006; Kray et al., 2008; Kray and Ferdinand, 2013; Lucenet et al., 2014; Kray et al., 2015). Recently, Doebel et al. (2018) showed that providing 4–5 year olds with familiar labels for new targets facilitates target tracking in a task involving random goal changes. The usefulness of verbal cueing has also been shown for improving behavioral regulation in the youngest children. Young children do not use these strategies spontaneously, but they can used them effectively, provided they have been trained to do so beforehand (Lucenet and Blaye, 2019). Most of these studies have shown less pronounced beneficial effects of the labeling strategy in children around 7–8 years of age (Kray et al., 2008; Kray and Ferdinand, 2013; Lucenet et al., 2014; Kray et al., 2015).

The pointing strategy is an external self-cueing strategy that involves touching or pointing to objects or targets with the fingertips. Through gesture, pointing enables to represent information that is not present in verbal representations, such as in holistic and visual imagery (Fletcher and Bray, 1996, 1997). The additional information provided by gestures sometimes represents more advanced knowledge than that represented verbally. In infants, communication enabling them to request information from others and the acquisition of different types of information involve pointing gestures, which are specific requests for labels (Lucca and Wilbourn, 2019). Gestural knowledge enables the youngest children to translate implicit information into a more explicit format, and may indicate a more advanced state of readiness of learning (O'Neill and Miller, 2013). Preschoolers especially rely on gestures such as pointing when faced with difficult cognitive tasks (O'Neill and Miller, 2013; Gordon et al., 2019).

Labeling and pointing strategies are thus useful to enhance the capacity of young children to gather relevant information, leading to correct identification of the goal of a task. The benefits of these self-cueing strategies have already been demonstrated in children aged 3 to 6 and in a variety of areas (attention task: Dusek, 1978; memory tasks: Fletcher and Bray, 1996, 1997; mathematical problem-solving task: Gordon et al., 2019). Moreover, self-cueing strategies, like many other cognitive strategies, should be trainable. A long tradition of research has established that cognitive strategies for remembering can be trained profitably (Bjorklund et al., 1997; Fletcher and Bray, 1997; Luxembourger et al., 2014). After training, a strategy is used with greater benefit, due to its greater effectiveness in the task. As to labeling and pointing, some work suggests that strategy training may help children learn to look for the most relevant perceptual cues (Yerys and Munakata, 2006; Kray et al., 2008; Kray and Ferdinand, 2013; Doebel et al., 2018; Fitamen et al., 2019; Lucenet and Blaye, 2019). Another point of interest lies on the possibility of these strategies to be transferred. However, researchers have not yet sought to test whether those strategies, once trained, can be transferred with benefit to a slightly different task. Yet, strategy transfer is core in developmental and educational psychology (Clerc et al., 2014).

Transfer is essential and omnipresent. To be able to generalize skills and use knowledge more flexibly, one needs to be able to transfer one’s learning (Brown, 1989). Transfer occurs when skills or knowledge acquired in a task are appropriately reused in a different, yet similar, task (Clerc et al., 2021). Depending on the degree of correspondence of the contexts, and of the content to be transferred between the learning task and the transfer task, transfer is considered as being either near or far (Barnett and Ceci, 2002). Near transfer is defined by the fact that the learning task and/or its context share several features with the transfer task and/or its context. Far transfer is defined by the fact that the learning task and/or its context share very few features with the transfer task and/or its context (Moser et al., 2015; Aladé et al., 2016). Task features refer to the knowledge domain (mathematics, physics, etc.), the materials used (text, picture, etc.), the procedures, the cover story, and the resolution principles. Contextual features refer to the physical environment, i.e., the location where the task is performed, as well as physical (Barnett and Ceci, 2002) or digital (Mombo et al., 2024) elements surrounding the task; and to the social environment, i.e., the people involved in performing the task (Klahr and Chen, 2011). As to strategy transfer, it requires identifying the relevant features that the transfer task shares with the learning task, in order to choose in one’s strategic repertoire the most relevant strategy for achieving the desired goal (Pressley and Harris, 2009). Studying self-cueing strategies seems particularly appropriate for measuring their effects on goal identification, both following training in a learning task and, in a transfer task. To the best of our knowledge, no study has addressed the issue of training and transfer of labeling and pointing strategies in 4 year olds.

The aim of this study was to test to which extent two goal self-cueing strategies—labeling and pointing—can help 4 year olds to spot relevant cues, which should enable them to clearly identify the goal of a task. We sought to verify whether explicit strategy training is beneficial in improving young children’s goal-identification performance, and whether such a benefit is maintained in a near transfer task. We hypothesized that training labeling and pointing would be beneficial, by facilitating to spot the relevant cues for goal identification. In the main task, trained children should produce the strategy more frequently than the nontrained ones (control group; H1a). They also should perform better in goal identification (H1b). In the transfer task, trained children should also perform better, both in strategy production (H2a) and in goal identification (H2b).

## 2 Materials and methods

### 2.1 Participants

Initially, 118 children aged of 4 years 6 months (*M* = 54 months, *SD* = 5.28 months) took part in the study. Their socio-cultural background was varied, as were parental socio-professional categories (mixture of disadvantaged, modest, and favored populations). All had French as a native language. These children were all enrolled in preschool classes located in the North of France in small to medium-sized towns. Of the 7 participating schools, 4 are classified as Priority Education Networks or equivalent, recognized as welcoming a socially mixed population. Teachers indicated that all the children in the sample were typically developing, with no specific cognitive or learning difficulties. Participants were assigned either to a first experimental groups (training in the labeling strategy) or to a second experimental group (training in the pointing strategy) or to a control group (no training). Age and Raven’s Progressive Colored Matrices (PM47) scores were used to ensure that the groups were homogeneous (see section 3.3 Group characteristics). The PM47 (‘CPM’; Raven, 1977) has been used to measure children’s intellectual performance. It is a reference psychometric test for assessing the g-factor and inductive ability of children aged 3 to l0 (Raven, 2000; Raven et al., 2003). It consists of three series of 12 boards, each illustrating a different lacunar figure. The child is asked to complete the figure by choosing one answer among six. The score corresponds to the number of correct answers on the 36 boards.

Participants in the study were faced with a card-sorting task. Correct sorting depended on a cue which was present on each card. To succeed in this task, the participant had to determine the sorting criterion, by identifying the cue that signaled the goal of the game: sorting according to shape or according to color. A first look at the strategic profiles shows that 12 participants were spontaneously strategic, i.e., they used the labeling strategy and/or the pointing strategy on their own prior to training (i.e., strategic score ≥ 9/16 during Pre-test; which shows an ability to use the strategy on more than half the trials. These children correctly sorted more than half of the cards, even if they carried the cues of the two criteria sorting: half color; the other half shape). When faced with this type of task, it is now well established that children up to the age of 5 tend to persist with the rules of the first sorting criterion (‘perseveration errors’; Simpson and Riggs, 2007; Blaye and Chevalier, 2011; Doebel and Zelazo, 2015). This represents 10.17% of the study population, which confirms that self-cueing strategies are rare in the strategic repertoire of 4 year olds. As the aim of the study was to test the impact of strategy training, it was not relevant to keep these spontaneously strategic children in our sample, so their scores were removed from the analyses. Furthermore, 7 additional participants were absent from at least one of the three sessions, which lead to the removal of their scores from the analyses. Therefore, we processed data from 99 children, aged 4 years 5 months (*M* = 54.1 months, *SD* = 5.32 months, 54 girls). These 99 participants were randomly assigned to one of the three groups.

### 2.2 Materials and measures

The task is made of two card-sorting games that we have specially created for the purpose of this study. We used commercially available decks of cards as a basis, to which we added visual cues to create the experimental material. Thus, no child has ever encountered the cards as presented here—in the butterfly study—before they were recruited in this study. The first game is the animal game, which includes cards from the game COCOTAKI (Gigamic^®^). The cards represent four different animals (e.g., sheep, cow, dog, cat) in four colors (e.g., red, green, yellow and blue). The second game is the shape game, which includes cards from the game COLOR ADDICT KIDZ (Drôles de jeux!^®^). The cards represent four different shapes (e.g., square, heart, triangle, round) in the same four colors as in the first game (e.g., red, green, yellow and blue) (see Supplementary Material A). For the purpose of the study, a small butterfly picture was added to each card, as a visual cue symbolizing the sorting criterion. Depending on the card, the butterfly comes in two formats: a gray butterfly symbolizes the “animal” (or “shape,” see below) sorting criterion, and a colored butterfly symbolizes the “color” sorting criterion. Boards illustrating the sorting rules for butterfly cues have been created to identify the goal of the task, and will henceforth be called illustrated cue boards (see Supplementary Material B). These boards are used to indicate the current sorting rule, and are intended to be available on each trial for all children and in all phases. Four different sets of 16 cards were composed to enable each child to receive its own set during the collective training phase. In each set of cards, eight cards contain a gray butterfly and eight contain a colored butterfly. The type of butterfly (gray vs. colored) is the only difference between the two subsets of eight cards, which are strictly identical otherwise.

The main task consists of a three-stage card-sorting game: two parts of cards to be sorted according to a single specific criterion—a color part, then an animal part—each with six cards to be sorted, and a final mixed part with 16 mixed cards (8 colored butterfly cards and 8 gray butterfly cards) where the two sorting criteria alternate randomly. There is no control over the order in which the cards appear, as in a classic card game where the 16 cards are shuffled completely at random. The principle of the game is to sort the cards according to a criterion (color or animal). Each participant must place the card in the corresponding box on the grid provided (see Supplementary Material C). The grid is placed in front of the child during the individual phases, and in the center of the table during the collective training phase. In each phase, the child must sort six cards twice in the single-criteria games (color, then animal), and 16 cards in the mixed game.

The transfer task is identical to the main task, excepted that the cards used depict shapes instead of animals (e.g., heart, triangle, circle, square). Half participants received animal cards in the main task and shape cards at transfer, and the reverse for the other half.

### 2.3 Procedure

The school district gave its authorization and written consent was given to the parents, and children gave oral consent to participate and signed a consent form by circling the corresponding smiley. The ethics committee in behavioral sciences of the University of Lille validated the protocol.

The study comprises four successive phases: (1) Pre-test; (2) Collective training in the use of a strategy; (3) Post-test; (4) Transfer. These steps are listed in Table 1. Each step is described in greater detail below.

**Table 1:** Typical study steps.

Task	Indexed sorting task			
Day	Monday	Tuesday	Thursday	Friday
Step	1. Pre-test	2. Training	3. Post-Test	4. Transfer
Material	**Total: 28 trials** Animal cards 6 cards with gray butterfly 6 cards with colored butterfly 16 mixed cards 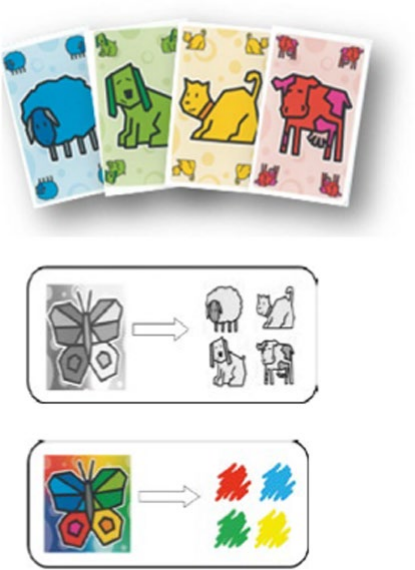	**Total: 28 trials** Animal cards 6 cards with gray butterfly 6 cards with colored butterfly 16 mixed cards 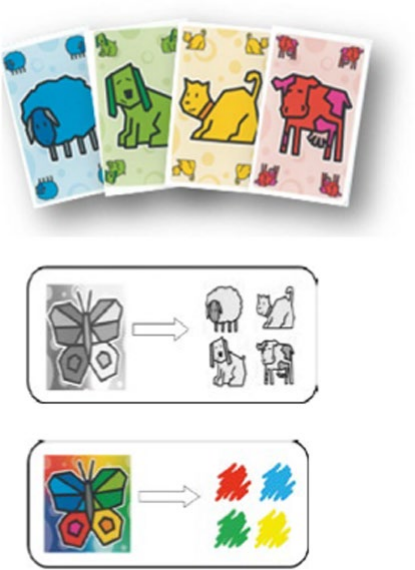	**Total: 28 trials** Animal cards 6 cards with gray butterfly 6 cards with colored butterfly 16 mixed cards 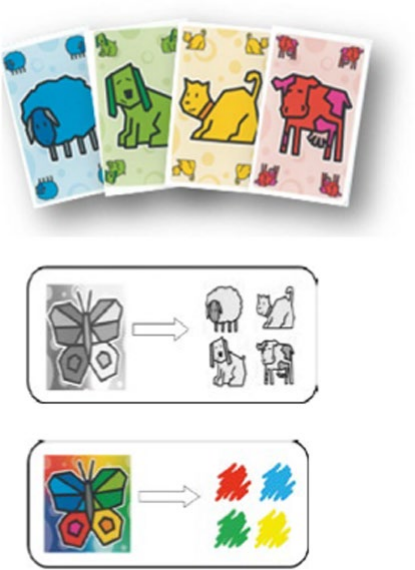	**Total: 28 trials** Shapes cards 6 cards with gray butterfly 6 cards with colored butterfly 16 mixed cards 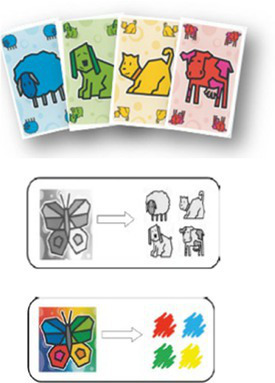
Score Statement	Mixed games Goal Score: number of correctly sorted cards /16 Labeling Strategy Score: number of times the strategy is used to sort the cards /16 Pointing Strategy Score: number of times the strategy is used to draw cards /16			

Beforehand, in order to reflect general intellectual functioning and create homogeneous groups, participants completed Raven’s PM47s.

***Pre-test***: the Pre-test consists of the child completing the three card-sorting parts (color; animals; mixed) of the game individually and without feedback. The color sorting grid is placed in front of the child, so that he can easily reach it. This phase begins with presenting the materials and checking that the child knows the colors: “*You see here, there are four colors: red, blue, yellow, green*”; then the cards “*Here, I have some cards. You see the same colors: there are red, blue, yellow and green cards, and there are also different animals in these colors: a sheep, a cat, a dog, and a cow. And look, on all the cards there’s also a colored butterfly stuck on them*.” The first part consists of playing the color game: “*We’re going to play a game. It’s called the color game. In the color game, all the reds go here, the blues go here, the yellows go here and the greens go here*. [The card which is used for the demonstration is placed in the corresponding box in the sorting grid] *To help you remember, there is this colored butterfly on the card, remember?”* [The butterfly on a card is shown and the illustrated cue boards (see Supplementary Material B; colored butterfly = color game) is presented]. “*I am going to leave this board that reminds you of the rule, look: colored butterfly, we are playing the color game*.”

A demonstration is given by the experimenter with a card from a batch other than the participant’s one, then the child also tries it out to see if he/she has understood the rules. They receive corrective and informative feedback: “*Have you understood? Let us give it a try. Take this card, where does it go?”* If the child places the card correctly, he/she is congratulated: “*Very good. You know how to play the color game. You have put the red card in the red box*.” If the child sorts incorrectly, he/she is corrected: “*No, in the color game, it is a red, so it has to go there, in the red box*.” After this trial run, the first game with six cards begins. The child receives his/her own batch of cards and has to sort them correctly. “*Now you have to put all these cards in the right place, but you do it yourself, I will not say anything! Are you ready?.”* No further instructions or feedback are longer provided.

On each trial, the goal score is recorded, consisting of the number of correctly sorted cards. The strategic score is also recorded, consisting of whether the child spontaneously uses one of the two strategies (labeling or pointing) for each sorted card.

Once the first part has been completed, comes the second part, in which the child must sort according to the second criterion: “*Great, you have done well with the color game. Now we are going to play a new game. We are not going to play the color game anymore. We are going to play the animal game*.” The second grid is put on the table instead of the first. “*In the animal game, all the WWs go here, the XXs here, the YYs here, and all the ZZs go there. To help us remember, there is still the butterfly on the card, but this time, look, it is gray!.”* The gray butterfly is shown on a card and, as in the previous part, the illustrated cue boards (gray butterfly = animal game) is presented and left visible on the table. A familiarization trial is carried out by the child, who receives feedback for this trial only. Then, he/she begins to sort the six new cards containing the gray butterfly but no feedback or instructions are longer given. As in the first game, the goal and strategy scores are recorded for each trial.

Once the second part is over, the third and final part is launched. “*Great, you have done well with the animals. Now I have got a slightly harder game for you. In this game, we mix up the two butterflies! Sometimes there are cards with the colored butterfly like this [*show 1 card*] and so that means you have to play the color game. And sometimes there are cards with the gray butterfly like this [*show 1 card*] and that means you have to play the animal game. Right? Here we go*!” This last mixed game involves 16 cards to be sorted—eight with gray butterflies and eight with colored butterflies—which are randomly shuffled as in a classic card game. As in the previous parts, goal score and strategy score are recorded for each sorted card.

***Training***: the collective training phase is carried out in small groups of four children, who are encouraged to use the trained strategy of self-identification of the goal (e.g., labeling or pointing, depending on the group) each time they have to lay down a card. Each group of 4 children is divided into pairs, each of which is given a sorting grid to share and a common set of cards. This set comprises 32 cards to be sorted, so that each child always has 16 cards to sort. After a reminder of the two sorting rules, each child in turn puts down his/her card on the table and the mixed game is launched. When the first child in the pair has put down its card, the second child (the one not playing at the time) is responsible for ensuring that the strategy is used and that the answer is correct. He/she gives feedback to the player. The roles are reversed for the next card, and so on with all the cards to be sorted for the game. In the goal labeling strategy training condition, the child must name the current sorting criterion, which is determined by the cue visible on the card (colored butterfly = color game, gray butterfly = animal game), before placing the card on the grid. In the goal pointing strategy training condition, the child must point one of the two illustrated cue boards which are placed below the sorting grid and easily accessible to the child. In both training conditions, feedback on both the quality of the answer (correct/incorrect) and of the justification (criterion in progress) is given systematically during this phase. For example, if the child places the card correctly, the experimenter and/or the second child in the pair validates the trial: “*You have used strategy X correctly and you have placed the red card in the red box*.” If the child does not use the strategy, he/she is reminded to do so. If he/she sorts incorrectly, he/she is said “*No, it is a red one, so it must go there in the color game*.” In the active control group, the children play in pairs, but without any further explanation or mention of strategies. “*Today we are going to play cards again, but this time we are going to play as a team!.”*

***Post-test***: during the Post-test, children are met again individually and with the same set of cards as in the Pre-test. The three parts are repeated (6 cards to be sorted according to animal criteria, 6 according to color criteria, 16 mixed cards), for a total of 28 cards to be sorted. No feedback is given.

***Transfer***: new cards from the other game (e.g., shape cards) are presented to the child. The procedure is the same as in Pre- and Post-test. The child must sort 28 cards through the three parts (6 color, 6 shape, 16 mixed). The new deck of cards is presented, and it is ensured that children know the shapes present on the new cards: “*Today we are going to play card games one last time, but look, these are new cards*!.” No attempt is made to demonstrate. Instructions are given for each game, but no indication of strategy or feedback is given.

In short, all children go through the four phases of the experiment in the same order: Pre-test/Training/Post-test/Transfer. Only the order of the decks used as main and transfer tasks is counterbalanced. Thus, half the participants completed the Pre-test, Training and Post-test phases with cards from the “Animal” deck, and the Transfer phase with cards from the “Shape” deck; for the other half it was the other way around. The same set of cards is used in each phase for a given child. In each phase, the participant must sort a total of 28 cards. Furthermore, in the first two parts in a phase (sorting on a single criterion), the child must sort the same six cards according to the associated visual cue: the gray butterfly for animal game and the colored butterfly for color game. During the third part in a phase (sorting on both criteria), the 16 cards are grouped together and must be sorted following both criteria alternatively. The correctly sorted cards constitute the goal score. In total, each child performs 28 trials × 3 phases (Pre-test, Post-test, Transfer) + 16 training trials = 100 trials.

## 3 Results

### 3.1 Experimental design and dependent variables

A 3-group (labeling, pointing, control) × 3-phase (Pre-test, Post-test, Transfer) experimental design was used. Several measures were used as dependent variables. The Raven Progressive Color Matrix (PM47) score was used to assess general intellectual ability. This score can vary from 0 to 36. A goal score was used, reflecting correct identification of the goal of the task. Only the mixed trials were considered to compute this goal score, as these are the only ones during which the participant had to determine the sorting criterion. Cards with the two cues appeared totally random. Each trial during which the goal is correctly chosen is credited one point. This score can thus vary from 0 to16. The use of labeling and pointing strategies in each mixed trial was also recorded, and determined the strategic scores, which can also reach a maximum of 16. Both strategy scores and goal score were recorded for the 48 mixed trials (16 trials × 3 phases). Descriptive statistics for these variables are summarized in Table 2. These analyses were carried out using the JAMOVI^©^ software.

**Table 2:** Descriptive statistics of the different variables related to the card-sorting task (*n* = 99) by groups.

				Pre-test			Post-test			Transfer		
	Group	Age	PM47	Str Labeling score */16*	Str Pointing score */16*	Goal score */16*	Str Labeling score */16*	Str Pointing score */16*	Goal score */16*	Str Labeling score */16*	Str Pointing score */16*	Goal score */16*
*Mean*	0	53.4	12.7	0.276	0.00	9.90	1.62	0.414	11.1	1.38	0.138	12.0
	1	54.5	12.6	1.40	0.00	10.9	8.20	0.714	14.0	8.00	0.543	14.0
	2	54.3	13.1	1.00	0.314	11.2	1.09	4.77	14.9	1.54	4.51	14.6
*SD*	0	6.33	5.27	0.922	0.00	3.67	4.07	2.04	4.55	3.89	0.743	4.19
	1	4.42	5.28	2.35	0.00	3.50	6.58	2.93	2.99	6.95	2.72	3.63
	2	5.33	5.77	2.00	1.02	3.36	3.51	6.72	2.20	3.94	6.53	2.70
Minimum	0	44	3	0	0	4	0	0	0	0	0	0
	1	47	3	0	0	2	0	0	6	0	0	0
	2	46	6	0	0	4	0	0	8	0	0	7
Maximum	0	65	23	4	0	16	16	11	16	16	4	16
	1	64	24	8	0	16	16	16	16	16	16	16
	2	65	29	7	5	16	16	16	16	16	16	16

Group 0: control group, *n* = 29; group 1: labeling group, *n* = 35; group 2: pointing group, *n* = 35.

### 3.2 Preliminary analyses

No hypotheses were made regarding the effect of gender or material on goal identifying (card sorting) and strategic performance. First, we wanted to ensure that these factors had no significant effect. Moreover, given that participants were faced with a first deck of cards during Pre-test / Training and Post-test, and a second deck of cards during Transfer, we wanted to check that both decks were of equivalent difficulty. We carried out a preliminary analyze. To minimize the risk of type 2 error, a multivariate analysis of covariance (MANCOVA) was preferred since it increases the statistical power. A MANCOVA on the factors 2 (Gender) × 2 (Deck of cards: Animal vs. Shape) was performed on goal scores, strategic labeling scores, and strategic pointing scores. No significant effects emerged, allowing us to ignore these factors in the remaining analyses [Gender: Wilk’s Lambda = 0.95, *F*(9,87) = 0.54, *p* = 0.84, *ns;* Deck of cards: Wilk’s Lambda = 0.88, *F*(9,87) = 1,37, *p* = 0.21, *ns*; Gender*Deck of cards: Wilk’s Lambda = 0.92, *F*(9,87) = 0.80, *p* = 0.61, *ns*].

### 3.3 Group characteristics

To check that the three groups were comparable at the outset in terms of age and intelligence (PM47), a MANOVA was carried out on these two variables. No significant difference appeared between groups on age [*F*(2, 98) = 0.36, *p* = 0.70, *ns*, *η^2^p* = 0.007] or on PM47 [*F*(2,98) = 0.07, *p* = 0.93*, ns*, *η^2^p* = 0.002]. Thus, these two factors were not included in subsequent analyses, and the three groups are considered to be equivalent. The Table 2 shows the characteristics of the groups and their distribution.

### 3.4 Strategy training and goal identifying

**Figure 1:**
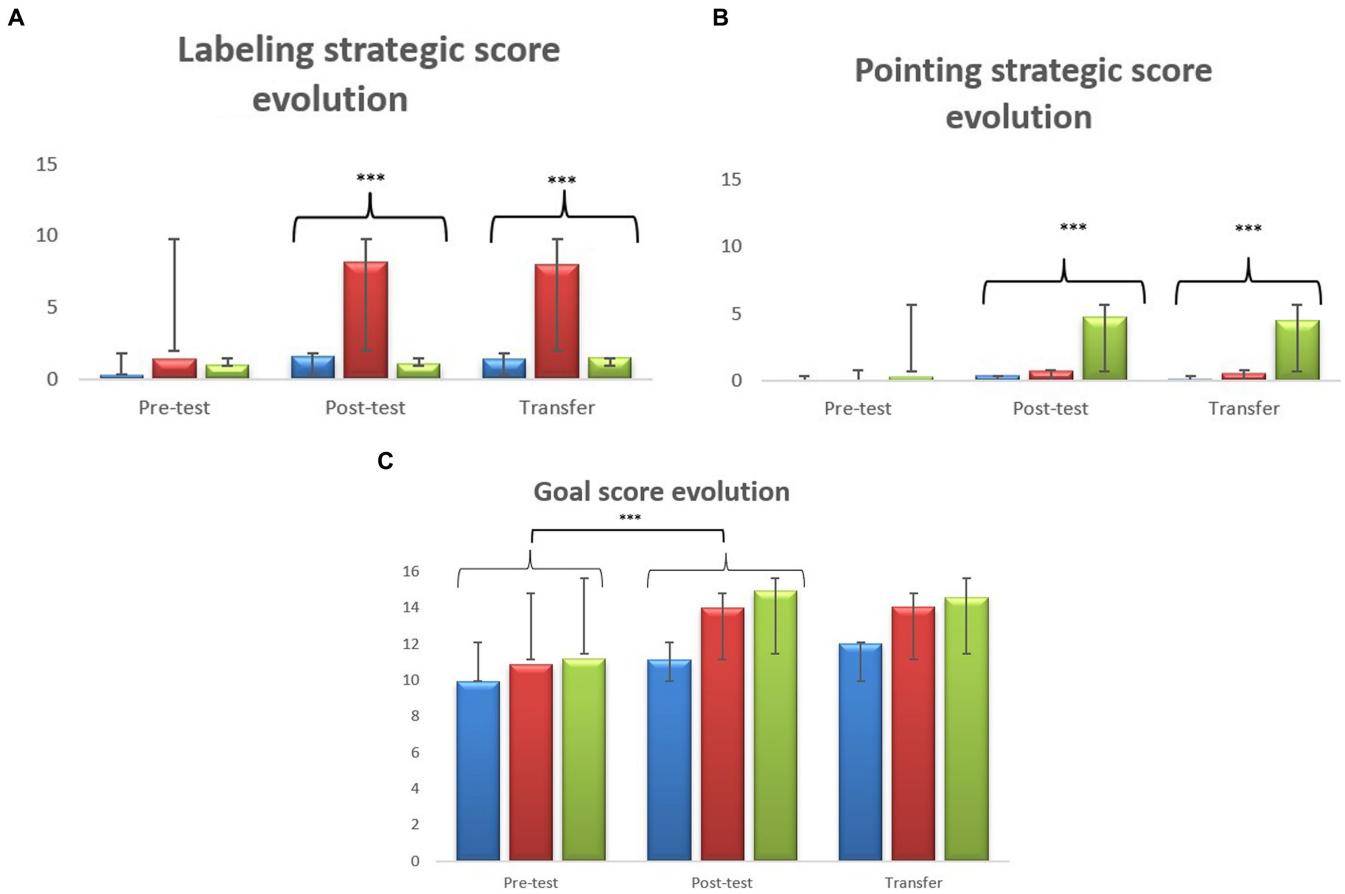
The three mean scores at pre-test, post-test and transfer phases, in two trained groups (labeling and pointing) and untrained group (control). **(A)** Mean labeling strategic scores. **(B)** Mean pointing strategic scores. **(C)** Mean goal score. Vertical bars indicate standard deviation, significance levels: ***p < 0.001.

Descriptive statistics are summarized in Table 2. Figure 1 shows the evolution of the three scores over the three Pre-test/Post-test/Transfer phases, whatever the group.

In order to gain a fine-grained understanding of the evolution of the three different scores in the three groups, children’s performance was analyzed according to the group they belonged to and to the three experimental phases. Three analyses of variance (ANOVAS) 3 Groups (trained labeling; trained pointing; untrained) × 3 Tasks (Pre-test, Post-test, Transfer) with repeated measures on the last factor were performed, one for each dependent variable (labeling, pointing, goal scores).

### 3.5 Labeling strategy

The main effect of Task was significant, *F*(2,192) = 28.8, *p < *0.001, *η^2^_p_* = 0.230. Bonferroni tests showed significant better results at Post-test (*M* = 3.635, *SE* = 0.50) when compared to Pre-test (*M* = 0.892, *SE* = 0.192), *p* < 0.001. No significant differences appeared between Post-test and Transfer. The main effect of Group was significant, *F*(2,96) = 19.8, *p < *0.001, *η^2^_p_* = 0.292. Bonferroni tests showed better results for the labeling group (*M* = 5.87; *SE* = 0.602) when compared to both the control group (*M* = 1.09; *SE* = 0.661), *p* < 0.001 and the pointing group (*M* = 1.21; *SE* = 0.602), *p* < 0.001. No significant difference appeared between the pointing group and the control group. The Task * Group interaction was significant, *F*(4,192) = 16.1, *p < *0.001, *η^2^_p_* = 0.251. Bonferroni tests localized the significant differences. The three groups did not differ significantly at Pre-test, which confirms that they were comparable at the beginning of the study. At Post-test, the labeling score of the labeling group (*M* = 8.2; *SE* = 0.837) differed significantly from those of the pointing group (*M* = 1.086; *SE* = 0.837), *p* < 0.001, and the control group (*M* = 1.621; *SE* = 0.919), *p* < 0.001. No significant difference appeared between the pointing group and the control group. At Transfer, results of the labeling group (*M* = 8; *SE* = 0.879) differed significantly from those of the pointing group (*M* = 1.543; *SE = *0.879), *p* < 0.001, and the control group (*M* = 1.379; *SE = *0.966), *p* < 0.001. No significant difference appeared between the pointing group and the control group. Lastly, the labeling group was the only one showing significantly increased performances from Pre-test (*M* = 1.40; *SE* = 0.322) to Post-test (*M* = 8.20; *SE* = 0.837), *p* < 0.001. No other significant effect was found. Of interest here is that no significant differences appeared from Pre-test to Post-test in the pointing group and in the control group.

### 3.6 Pointing strategy

The main effect of Task was significant, *F*(2,192) = 11.95, *p < *0.001, *η^2^_p_* = 0.111. Bonferroni tests showed better results at Post-test (*M* = 1.967, *SE* = 0.454) when compared to Pre-test (*M* = 0.105, *SE* = 0.061), *p* < 0.001. No significant differences appeared between Post-test and Transfer. The main effect of Group was significant, *F*(2,96) = 13.2, *p < *0.001, *η^2^_p_* = 0.215. Bonferroni tests showed better results for the pointing group (*M* = 3.20, *SE* = 0.453) when compared to both the control group (*M* = 0.184; *SE* = 0.498), *p* < 0.001 and the labeling group (*M* = 0.419; *SE* = 0.453), *p* < 0.001. No significant difference appeared between the labeling group and the control group. The Task * Group interaction was significant, *F*(4,192) = 6.69, *p < *0.001, *η^2^_p_* = 0.122. Bonferroni tests localized the significant differences. The three groups did not differ significantly at Pre-test, which confirms that they were comparable at the beginning of the study. At Post-test, the pointing score of the pointing group (*M* = 4.771; *SE* = 0.761) differed significantly from those of the labeling group (*M* = 0.714; *SE* = 0.761), *p* < 0.01, and the control group (*M* = 0.414; *SE* = 0.836), *p* < 0.01. No significant differences appeared between the labeling group and the control group. At Transfer, results of the pointing group (*M* = 4.514; *SE* = 0.714) differed significantly from those of the labeling group (*M* = 0.543; *SE* = 0.714), *p* < 0.01, and the control group (*M* = 0.138; *SE* = 0.785), *p* < 0.01. No significant differences appeared between the labeling group and the control group. Lastly, the performances of the pointing group increased significantly from Pre-test (*M* = 0.314; *SE* = 0.103) to Post-test (*M* = 4.771; *SE* = 0.761), *p* < 0.001. No other significant effect was found. Of interest here is that no significant differences appeared from Pre-test to Post-test in the labeling group and in the control group.

### 3.7 Goal score

The main effect of Task was significant, *F*(2,192) = 39.11, *p < *0.001, *η^2^_p_* = 0.289, and Bonferroni tests showed better result at Post-test (*M* = 13.4, *SE* = 0.333) when compared to Pre-test (*M* = 10.6, *SE* = 0.353), *p < *0.001. No significant differences appeared between Post-test and Transfer. The main effect of Group was significant, *F*(2,96) = 7.30, *p < *0.001, *η^2^_p_* = 0.132. Bonferroni tests showed significantly better goal scores for the labeling group (*M* = 13; *SE* = 0.461), when compared to the control group (*M* = 11; *SE* = 0.506), *p < *0.017. This was also the case for the pointing group (*M = *13.6; *SE* = 0.461), when compared to the control group, *p* < 0.001. No significant difference appeared between the two trained groups. The Task * Group interaction effect was not significant.

**Figure 2:**
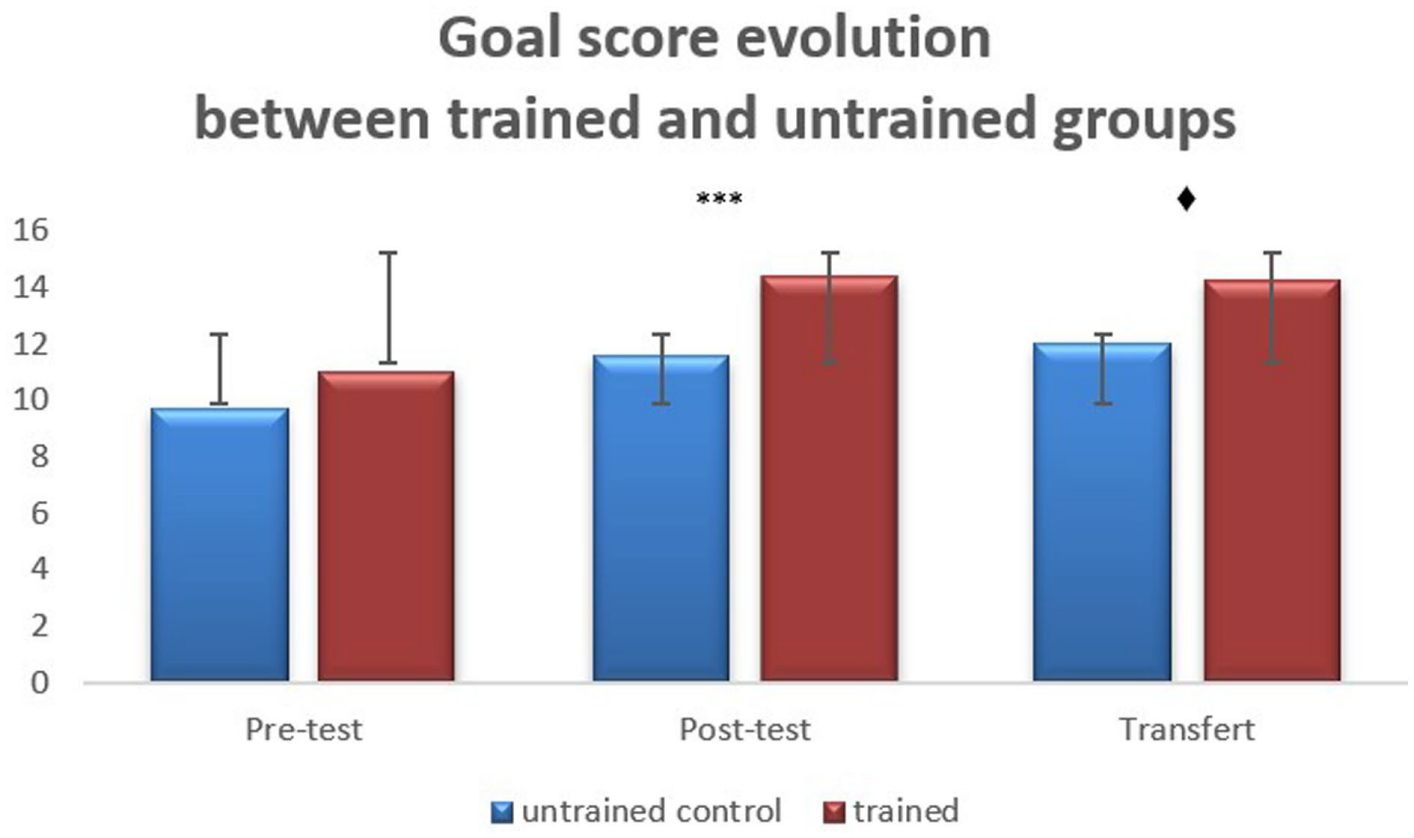
The evolution of goal scores at pre-test, post-test and transfer phases, in trained children and control children (untrained). Vertical bars indicate standard deviation, significance levels: ***p < 0.001, ♦ significant trend, 0.05 < *p* < 0.10.

In order to better appreciate the progression of the goal scores in the three groups between Pre-test and Post-test, effect sizes were calculated using Cohen’s *d* (Cohen, 1988; Wilkinson, 1999). They are large for both trained groups (labeling group *d* = 0.97; pointing group *d* = 1.32) and small for the control group (*d* = 0.30). Furthermore, as the two trained groups showed no significant differences between them, we gathered their performances in order to obtain a single trained group that we compared to the control group. Such an analysis may inform globally on the beneficial effect of self-cueing strategy training on using available relevant cues for identifying the goal of a task. Figure 2 shows the goal scores of this new single trained group as opposed to the untrained group, over the three Pre-test/Post-test/Transfer phases.

An analysis of variance (ANOVA) 2 groups (trained vs. control) × 3 Tasks (Pre-test, Post-test, Transfer) with repeated measures on the last factor was performed on goal scores. The main effect of Task was significant, *F*(2,194) = 27.02, *p < *0.001, *η^2^_p_* = 0.218, and Bonferroni tests showed that the goal score improved significantly between Pre-test (*M = *10.5; *SE* = 0.385) and Post-test (*M* = 12.8; *SE = *0.365), *p < *0.001. No significant difference appeared between Post-test and Transfer. Of greatest interest here are the effects including the group factor. The main effect of Group was significant, *F*(1,97) = 13.8, *p < *0.001, *η^2^_p_* = 0.125, better result being exhibited by the trained group (*M = *13.3; *SE* = 0.326) when compared to the control group (*M* = 11; *SE* = 0.506). The Task * Group interaction was also significant, *F*(2,194) = 3,79, *p < *0.05, *η^2^_p_* = 0.038. Bonferroni tests localized the significant differences. As we were only interested in the three inter-group comparisons, one for each task, the relevant *p* value for the Bonferroni test was set at 0.05/3 = 0.017. The two groups did not differ significantly at Pre-test. At Post-test, the trained group (*M* = 14.46; *SE* = 0.396) significantly outperformed the control group (*M* = 11.14; *SE = *0.615) on goal identification, *p* < 0.001. At Transfer, a significant trend can be noted between the trained group (*M* = 14.30; *SE = *0.419) and the control group (*M* = 12.03; *SE = *0.651), *p = *0.064. The calculated effect size between both groups at Transfer was large (*d* = 2.27).

In summary, the strategy scores (labeling and pointing) changed over the course of the tasks and between the three groups. Participants trained in the use of a specific strategy used it more frequently than the untrained ones, at Post-test and at Transfer. The performances of goal identification changed over the course of the tasks: all three groups showed a significant improvement from Pre-test to Post-test. Moreover, a significant trend appeared at transfer between trained participants considered as a whole group and untrained participants.

## 4 Discussion

The primary aim of this study was to test the effect of training two strategies of self-cueing (e.g., labeling and pointing) in 4 year old spontaneously nonstrategic children. We hypothesized that trained children would perform better than untrained ones in producing the strategy, in a main task and in a transfer task. Furthermore, the use of these strategies, as a help to notice relevant cues allowing goal identification, was especially scrutinized. To this aim, not only strategy scores but also goal identification scores were measured, for which the hypothesis was made of better performances in trained participants, both in the main and in the transfer task.

### 4.1 Strategy use

Our first hypothesis was on the beneficial effect of training, on the ability of initially nonstrategic children to produce one or the other self-cueing strategy spontaneously after training. This concerns primarily the main task, which was used here in the Pre-test, training, and Post-test phases. This hypothesis was validated, since trained children scored higher than untrained ones at Post-test. Indeed, with regard to strategy scores, considering each task and each group separately, several significant effects in favor of the two trained groups were observed. The labeling strategy trained group produced the labeling strategy more at Post-test than both the pointing group and the control group. A similar pattern was found for the pointing strategy trained group, that produced the pointing strategy more at Post-test than both the labeling group and the control group. Thus, children trained to produce a self-cueing strategy, being labeling or pointing, seem to be sensitive to training, by spontaneously producing the trained strategies at Post-test more frequently than untrained children. The benefits of these two self-cueing strategies have already been studied in young children. Research had shown that these two self-cueing strategies enable children aged 3 to 6 to search for the most relevant perceptual cues to solve a task (Yerys and Munakata, 2006; Kray et al., 2008; Kray and Ferdinand, 2013; Doebel et al., 2018; Fitamen et al., 2019; Gordon et al., 2019; Lucenet and Blaye, 2019). However, to date, no study investigated the possibility of training children in these strategies. The present study adds important results to this field of research, by showing that strategy training is effective. Indeed, training 4 year olds in the labeling strategy and in the pointing strategy enabled participants to make better use of the relevant cues necessary to identify the goal of a task. This extends previous research by showing a positive effect of training.

A positive effect of training, on the acquisition of diverse cognitive strategies in young children, has been emphasized by several researchers, particularly with regard to memorization strategies (Schneider and Ornstein, 2019) and problem-solving strategies (Bielaczyc et al., 1995; Stad et al., 2017). This has been much less studied regarding goal self-cueing strategies. The present study allows us to provide some clarification on the use of these two strategies in 4 year olds. At this age, motor skills, as well as language skills, are far from being fully developed. Strategies based on motor skills (e.g., pointing) and on language (e.g., labeling) may be difficult for such young children, making any benefit of strategy training uncertain. Positive effects of training 4-year-old children in pointing and labeling were observed here. Thus, our results provide evidence of the efficiency of explicit training on the use of two self-cueing strategies. These two strategies help children to notice relevant cues by themselves, with little or no help. These are useful strategies to become more autonomous in the search for such cues. Thus, efficient strategy training contributes to help children develop skills for relevancy. In addition, most studies on these two strategies have been conducted within the theoretical framework of cognitive control, primarily focusing on the benefit caused by the use of these strategies rather than on the use of the strategies *per se*. Our results provide further explanations within the SRL framework.

One of the specific features of SRL is that it introduces a processual perspective on learning, making it useful framework (Efklides, 2011; Winne, 2018). SRL is seen as a cyclical process. It is self-initiated by the learner, who must represent the task, plan how to carry it out, monitor and evaluate its execution and, finally interpret its performance, all while adapting to contextual elements (e.g., possible difficulties and emotions). SRL manifests itself in the use of cognitive strategies that enable the learner to achieve the desired goal. It involves a repertoire of effective strategies for learning. One of the major challenges is the effectiveness with which learners select, combine and coordinate these strategies (Boekaerts, 1999). These strategies must have been learned and tested in various situations in order to assess their validity (Winne, 1996; Winne and Perry, 2000). Thus, SRL develops through experience and teaching, following multiple exposures to strategies and explanations about the conditions of their efficient use (Winne, 1996). If teaching, and practicing occasions, provide opportunities to learn where and when strategies are efficient, children will more likely continue to use them, as well as transfer them to new tasks (O'Sullivan and Pressley, 1984; Pressley and Harris, 2009). The results of the present study are in line with this, showing for the first time that self-cueing strategies are trainable and transferable in 4-year-old children. Strategy training allowed participants to (1) clearly identify the goal of the task, which was here to select the right sorting criterion among two possible criteria, and which corresponds to the first step of SRL and, (2) use this strategy efficiently by succeeding in the card-sorting game, which corresponds to the second step of SRL. Another point of interest lies on the possibility of these self-cueing strategies to be transferred. This is what we specifically set out to explore in this study.

### 4.2 Strategy transfer

Our second hypothesis was on the ability of initially nonstrategic children to transfer, on new material, the trained self-cueing strategy. The results show that trained children not only improved their strategy score significantly from Pre-test to Post-test, but also maintained their higher level of strategy score in the transfer task where new cards were introduced. This hypothesis was thus also validated, which has important implications regarding strategy transfer. Transfer situations constitute a valuable way of studying the processes at stake, when children judge the relevance of a piece of information to make the right decision in a task. Early work on SRL (Zimmerman, 1986, 1990; Winne, 1995) has shown that it is difficult, and quite rare, to transfer trained strategies to other contexts. While great attention has been paid to the persistence of benefits of strategy training in trained tasks, it has been less studied in transfer tasks.

In the present study, participants maintained their high strategy scores in the transfer task. These results may be due to an enhanced capacity of the trained children to adapt the strategies to the transfer task, due to training. In the main task, these children received explicit training in self-cueing strategies through either labeling or pointing. It has long been known that explicit training of cognitive strategies enables children to maintain strategies in a transfer task (Whittaker, 1988; Gentner et al., 2003; Clerc et al., 2021). Explicit strategy training is likely to reduce the cognitive cost associated with strategy production, due to the strategy being more automatized (Coyle and Bjorklund, 1997), which has been hypothesized to play a role in strategy transfer as well (Clerc et al., 2014). Although they are grounded in memory strategy research, these results may be easily applied to the present study. When facing new cards in the transfer task, trained children were likely to adapt the trained strategies with more ease than untrained ones. The high amount of training seems to be enough to help them automatize the production of the strategies, saving part of one’s cognitive resources to analyze the transfer task and its requirements. Admittedly, as the main and the transfer tasks were structurally similar, transferring the strategies was relatively easy, as can be deduced from strategy transfer by untrained (control) children as well. Given the many features shared by the main task and the transfer task in a near-transfer situation (e.g., domains, materials, procedures, etc.), transfer is generally successful (Holyoak and Koh, 1987; Aladé et al., 2016). Nevertheless, near transfer was still better in trained participants, attesting for the beneficial role of strategy training on strategy transfer. Consequently, strategy training revealed also beneficial for young children to choose relevant cues (e.g., sorting criteria) more frequently than untrained children, not only in the training task but also in the new, transfer, task. This was allowed by the extensive training that these children received.

A practical application of this study may be that when children learn a new strategy, they may need to practice it extensively. Indeed, it may be that only extensive practice, well beyond the time when children just seem to have acquired a strategy, is necessary for them to derive real and substantial benefit from it. Children need to be able to make sense of the task, determine what to do and how to do it, and to judge the relevance of the available information at the time they take any decision, all highly time-consuming processes. This is in line with recommendations from researchers in SRL, who point out that having a repertoire of strategies and being trained in their use is not enough. Learners must learn to make personal choices according to context and conditions (Miele and Scholer, 2018; Winne, 2018; Leclercq et al., 2023). To achieve this, strategies need not only to be learned, but also to be experimented and practiced repeatedly in a variety of situations, allowing to assess their validity and conditions of use (Winne, 2011, 2018). Such repeated extensive practice can also be used to identify the goal of the task, the second focus of our study.

### 4.3 Goal identification

Our third and fourth hypotheses were that trained children would perform better at identifying the goal of the task (e.g., sorting cards following a given criterion) than those in the control group. We assumed that this would be observed in both the main and the transfer tasks. This hypothesis was only partially validated. The trained groups performed significantly better than the control group overall, as evidenced by the main effect of group on goal identification. This gives evidence of a positive effect of self-cueing strategy on goal identification. However, this was not qualified by a significant group*task interaction, whereas the three groups performed equally well at Pre-test. This gives also evidence of a positive, albeit probably smaller, effect of mere practicing the task in the untrained participants. In other words, untrained children repeatedly exposed to the sorting task with the cues (gray or colored butterfly), and the illustrated cue boards, were also able to progress toward better identifying a goal. This is attested by the main effect of task, since the performance of all three groups improved over the course of the tasks, whether they were trained or not. This lack of significant difference between the groups in goal identification at transfer may reflect the difficulty of transfer. As mentioned above, the trained participants transferred the trained strategy. Yet, it seems that the transferred strategy had no beneficial impact on those participants’ goal-identification performance, since untrained participants performed equally well on goal-identification in the transfer task. Stated differently, the trained strategy was transferred but its benefit was not. It seems, thus, that strategy training was enough to ensure higher strategy transfer, but not enough for the benefit of the strategy to be transferred as well. Such a pattern of results was already found in analogical problem-solving strategy research with slightly older children. Indeed, two studies showed a benefit of training between Pre- and Post-test in 7-years-old children asked to solve isomorphic analogous problems. However, this benefit did not provide significant additional improvement when children had to construct analogous tasks in a transfer phase, since their performance was equivalent to that of untrained children (Resing et al., 2016; Vogelaar and Resing, 2018). Other studies showed that children trained in memory strategies do not systematically encounter a benefit from strategy training, either in the same task (Bjorklund et al., 1997) or in a transfer phase (Schwenck et al., 2007). In sum, the present study confirms research on so-called utilization deficiencies (Miller, 1990), that has long shown that strategy training can be beneficial for strategy use *per se*, but does not always translate into any strategy benefit in the task at hand (Bjorklund et al., 1997), including transfer tasks (Schwenck et al., 2007; Clerc et al., 2014).

A noteworthy point though, is that the difference between Pre- and Post-test was stronger in both trained groups than in the control group, as shown by the large effect sizes in both trained groups and the small one in the control group. Considered along with the absence of any effect of the group*task interaction, these results are puzzling and may be due to the too small sample size. Indeed, only 33 children composed each group, which may have been not enough to show the group*task interaction effect. We then gathered data from both trained groups in a single trained group including more participants and compared it to the control group. This new analysis allowed us to show that trained participants, considered as a whole group, performed better at goal identification than untrained ones. This was probably due to a gain in statistical power, that was permitted by gathering data from both labeling and pointing trained groups to compose a unique single trained group. Indeed, performance of this newly defined trained group was significantly better than those of untrained children at Post-test, attesting more strongly to the beneficial effect of self-cueing strategy training on goal identification. Moreover, a significant trend was now observed between trained and untrained children at transfer. This suggests that training young children in self-cueing strategies enhances their ability to adapt the strategies in a transfer task. Indeed, not only did the trained children correctly transfer the trained goal self-cueing strategy (e.g., transfer of the strategy *per se*), but it seems to have helped them identify the goal of the transfer task more efficiently than untrained children (e.g., transfer of the strategy benefit on goal identification).

Many researchers in the past argued that training may help children to make more efficient use of their cognitive strategies by reducing the cognitive cost associated to strategy use, in main tasks (Bjorklund et al., 1997) as in transfer tasks (Clerc et al., 2021). In this study, a self-cueing strategy produced with less effort, due to extensive training, may have required fewer cognitive resources, allowing children to dedicate more resources to properly identify the goal of the task. Our study therefore confirms the beneficial effect of explicit strategy training in children, and suggests that self-cueing strategy training can help children resist to the decrease of strategy efficiency at transfer that was observed in several studies (Blöte et al., 1999; Clerc et al., 2017, 2021). Admittedly, untrained children were also able to maintain in the transfer task the high level of goal identification that they exhibited at Post-test, speaking for a positive effect of mere repeated strategy use. However, if the significant trend between trained and untrained children observed at transfer would come to be confirmed in future studies, this would be additional evidence for the positive role of training self-cueing strategies in 4 year olds. Given that strategy transfer is difficult at this age, this is a promising result for any researchers interested in helping children to seek for any relevant cues necessary to identify the goal of a task. Being able to transfer one’s strategies in new tasks containing different materials means that children gain precious adaptation skills.

Our results extend the findings of Lucenet and Blaye (2019), who showed that encouraging children to construct an explicit representation of the goal of the task is essential for reinforcing cognitive control. In their study, they tested the impact of the same two self-cueing strategies (labeling and pointing) in children aged 5, 6 and 9. Moreover, their task was different from ours, and computerized. It was an adaptation of the cued task-switching paradigm (Meiran, 1996, cited in Lucenet and Blaye, 2019) with thirty-six drawings of colored animals: three different dogs and three different birds represented in three different shades of green and red. According to the signals, one must sort according to shape or color. In Lucenet and Blaye (2019)‘s study, the signal disappeared as soon as the stimulus was presented, and it was impossible to go back after the stimulus had been displayed. This maximizes the need to translate the signal into an explicit representation (Chevalier et al., 2017). They showed that, for the youngest two age groups (5 and 6 years), seeking an explicit goal representation before the next stimulus, whether verbal or pictorial, promoted goal identification. In other words, the use of goal self-cueing strategies such as labeling and pointing promotes success in this type of task. In our experiment, the average age of the children was 4.5 years, which is very young to be able to produce spontaneously and autonomously a self-cueing strategy, and benefit from it in goal identification. Our results provide empirical evidence of the value of these strategies in early childhood, as well as the benefit of training. Furthermore, our task was not computerized. It consisted in sorting cards, in the same way as in a conventional board game, which may be exploited further to conduct research on this topic in a more ecological context. Our results can also be interpreted by looking at the conditions and context in which the task is performed. These elements are important in the first phase of the SRL (Winne and Hadwin, 1998; Winne, 2018).

## 5 Limitations and perspectives

Although our results are informative regarding the effect of strategy training on the use of self-cueing strategies and on the ability to identify the goal of a task, this study presents some limitations. Only collective training was used here which may have had effects on the use of strategies in untrained children. Children in the control group may have benefited from so called co-regulation, which may explain their rather good performances in goal identification (Hadwin and Oshige, 2011; Hadwin et al., 2017). When interactions with peers are possible, the processes by which individuals regulate their activity are built collectively and involve questioning and reflection on their own resolution process. This may have been the case for the goals that children set for themselves or for the strategies that they used. Further studies should take this into account by contrasting collective vs. individual modes of training.

This study encourages the use of teaching practices such as explicit instruction of cognitive and metacognitive strategies. In concrete terms, this invites teachers to get young pupils to verbalize the goal of the task, and to question them on what they have understood about the goal of the activity to be carried out, as well as on how to achieve it. This may be done through explicit questions (eg. “*What do you have to do? How can you do it? What can help you*?”), in young children (Marulis et al., 2016) as well as in teenagers (Zohar and Ben-David, 2008). The results also show that it can be relevant to train children to use these two self-cueing strategies, and that it is therefore necessary to take the time to do this with the pupils. The aim is to help them identify the available cues—in the instructions, in the classroom, in the materials—to help them complete the task. This study also encourages teachers to involve students in the activity and share their ideas with them (Pino-Pasternak et al., 2014). Furthermore, it underlines the role of the classroom context and of the conditions for carrying out learning, two dimensions that gained momentum since the original work by Boekaerts and Corno (2005). Regarding training, the intensity of training, its collective *vs* individual nature, as well as the material used, all confirm the importance of context in strategy training, as was shown by developmental researchers (Bjorklund et al., 1997). In line with the young age of the participants in this study, collaborative self-regulation deserves also to be considered from an early age in the future (Hadwin and Oshige, 2011; Blair and Raver, 2015).

## 6 Conclusion

Helping learners to use efficient strategies to identify and achieve the goal of a task is important (Panadero, 2017; Winne, 2018). This allows them to judge the relevance of the information and what strategies to use. Thus, training self-cueing strategies that allow to achieve the goal of a task appears relevant (Fitamen et al., 2019; Lucenet and Blaye, 2019; Roebers et al., 2019). The present study aimed to better understand the impact of explicit training of two self-cueing strategies (labeling and pointing) on the ability of preschoolers to identify the goal of a task in two tasks (e.g., a main and a transfer task) and in an ecological context.

Training to use labeling and pointing strategies helped to increase the strategic scores and the goal scores. Our results thus support the recommendations provided by recent studies which suggest that cue utilization, as well as training to use strategies, can be promising avenues for promoting goal identification (Lucenet and Blaye, 2019; Roebers et al., 2019). This study shows that training benefits children aged 4, as had already been shown with computerized tasks (Doebel et al., 2018). These results provide new insights regarding the development of two distinct strategies, particularly involving language (labeling) or motor skills (pointing). Both strategies seem to help young children to become more able to notice the relevant cues necessary to identify the goal of a task. Extensive training of self-cueing strategies is a promising avenue for supporting the development of SRL and adaptation skills in young children.

## Data availability statement

The raw data supporting the conclusions of this article will be made available by the authors, without undue reservation.

## Ethics statement

The studies involving humans were approved by University of Lille Research Ethics Committee, France (2019-386-S77). The studies were conducted in accordance with the local legislation and institutional requirements. Written informed consent for participation in this study was provided by the participants’ legal guardians/next of kin.

## Author contributions

ML: Conceptualization, Formal analysis, Investigation, Methodology, Writing – original draft, Writing – review & editing. WTM: Writing – review & editing. JC: Supervision, Writing – review & editing.
