# Supplementary material for: Judgments of relevance in preschoolers: a study of training and transfer of self-cueing strategies
Source: Front Psychol. 2024 Jan 30;15:1341572. doi: 10.3389/fpsyg.2024.1341572 (PMC10863624; doi:10.3389/fpsyg.2024.1341572)

Supplementary Material

Supplementary Material A

THE DIFFERENT CARD SETS CREATED

Each child always has 2 x 8 cards of 4 categories in 4 colors: 1 set with a colored butterfly and 1 set with a gray butterfly

The card bases used for the Animals game are taken from the COCOTAKI game (each animal is represented in the four colors).

The card bases used for the Shapes game are taken from the COLOR ADDICT KIDZ game (each shape is represented in the four colors).


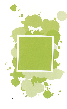


The different sets created for each game: Y = Yellow; B = Blue; R = Red; G= Green

| ***Animal game*** | **SHEEP**  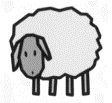 | | **CAT**  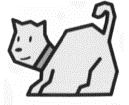 | | **DOG**  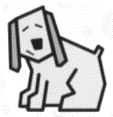 | | **COW**  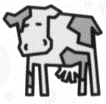 | | ***Shape game*** | 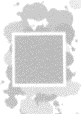**SQUARE** | | 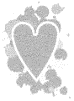**HEART** | | 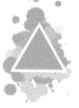**TRIANGLE** | | **CIRCLE**  **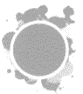** | |
| --- | --- | --- | --- | --- | --- | --- | --- | --- | --- | --- | --- | --- | --- | --- | --- | --- | --- |
| **set 1** | Y | B | Y | B | R | G | G | R | **Lot 1** | Y | B | Y | B | R | G | G | R |
| **set 2** | J | R | G | B | B | R | Y | G | **Lot 2** | Y | R | G | B | B | R | Y | G |
| **set 3** | B | G | Y | R | Y | G | R | B | **Lot 3** | B | G | Y | R | Y | G | R | B |
| **set 4** | G | R | R | G | Y | B | Y | B | **Lot 4** | G | R | R | G | Y | B | Y | B |

Supplementary Material B

ILLUSTRATED CUE BOARDS

Boards illustrating the sorting rules for butterfly cues

*Board for the Color Game*

**
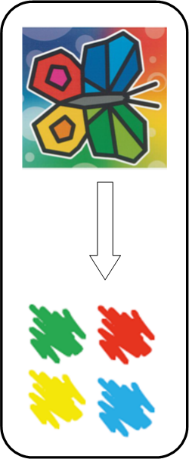
**

*Board for the Animal Game*

**
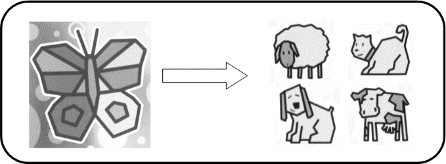
**

*Board for the Shape Game*

**
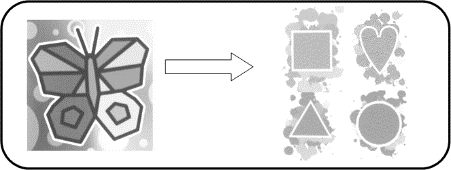
**

Supplementary Material C

CARD-SORTING GRIDS (available to children in A4 format)

*Grid 1 for the Animal Game*

Used in the Pre-Test, Training and Post-Test phases for the first half of participants. Used in the Transfer phase for the other half.


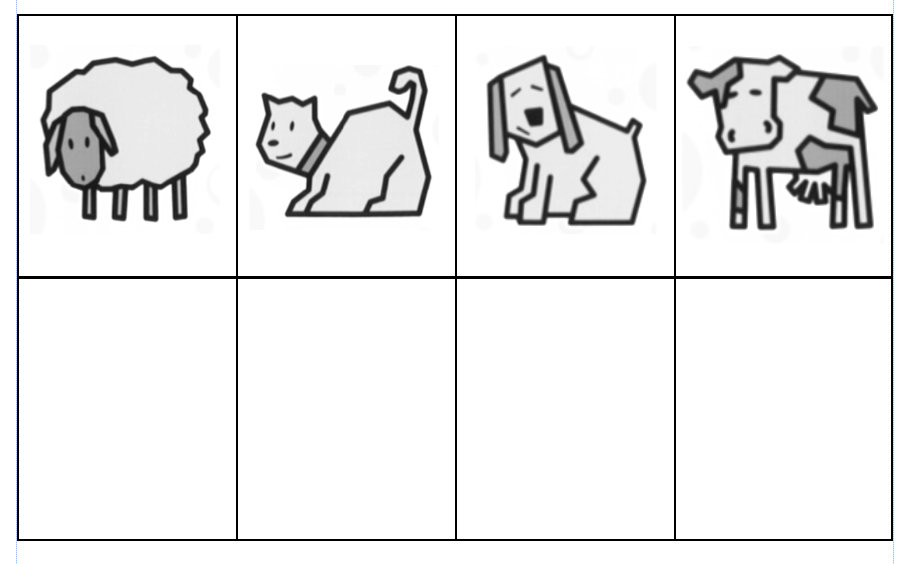


*Grid 2 for the Shape Game*

Used in the Transfer phase for the first half of participants; used in the Pre-Test, Training and Post-Test phases for the other half.


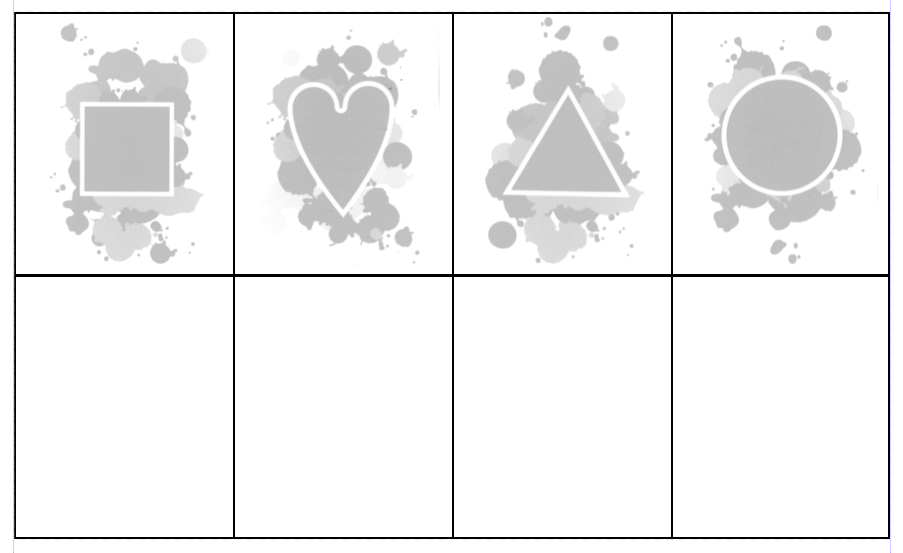


*Grid 3 for the Color Game*

Used for all Pre-Test, Training, Post-Test and Transfer phases and for all participants.


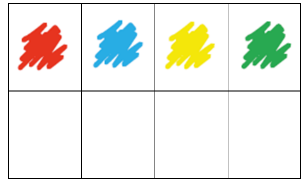

Supplement: Supplementary file 1 [file Data_Sheet_1.docx]
